# Dysmenorrhoea

**Published:** 1912-05

**Authors:** Stephen Y. Howell

**Affiliations:** A.M., M.D., M.R.C.S. England


### Dysmenorrhoea.

By STEPHEN Y. HOWELL, A.M., M.D., M.R.C.S. England

FROM puberty to the menopause, from the beginning of functional activity in the generative organs of the human female to its close, the disorders incident to menstruation may manifest themselves .

Influenced by climate, social environment and heredity, as well as by individual and racial peculiarities, the burden of this mysterious and altogether unesthetic handicap of womankind is borne through a variable part of her life. While the classical and generally accepted average bounds are the fourteenth and forty-fifth years, precocious infants and old women of eighty or more have alike nonplussed the medical fraternity by the regular recurrence of this mensal flow.

As is evidenced by its terminology, the periodicity of the discharge corresponds generally with the lunar cycle of twenty-eight, though ranging from twenty-four to thirty-four, days.

Concerning the correlation of ovulation and menstruation the opinions of many learned students of human and comparative physiology have clashed in wordy polemics during past years. At present, however, the dominant view favors their mutual independence. While the escape of the mature ovum from the Graafian vesicle and the phenomena of menstruation are usually synchronous, they may be, and usually are, of independent occurrence.

In these days, when the biological importance of the ductless glands is being so rapidly established, any facts, germane to our subject, which throw light upon the ovarian functions are full of interest. Those mysterious chemical messengers which are concerned in the regulation of somatic activities, aptly termed "hormones," are becoming, through the labors of Starling and others, more and more familiar. The *secretin* which abounds in the duodenal mucous membrane and which regulates, through the medium of the blood, the secretion of the pancreatic juice and bile proves to be but one of a probable many. Chemical substances secreted by the thymus, thyroid, parathyroid, suprarenal and hypophysial glands must be included in this list of hormones. The roles played by carbonic acid in the regulation of respiration, by gastrin in gastric digestion and by the hormonic material secreted by the developing ovum *in utero,* which passes into the blood-stream and incites the mammary activity incident to pregnancy, include these in the same category. In the case of the ovary likewise, evidence points to the presence of such a chemical messenger, as the following facts would seem to prove: As a rule, ovulation in lower mammals is associated with a state known as "heat," "rut" or "oestrus," preceding which is a stage termed "prooestrum," in which phenomena corresponding to menstruation appear. If the ovaries of such mammals are completely extirpated, heat and its menstrual accompaniments fail to recur. Should ovarian extract, prepared from the ovaries of an animal in the rutting state, be injected into the sterilized one however, the latter again becomes in heat.

The ovaries, tubes and uterus of a woman are congested and enlarged during the menstrual period. The mucous membrane becomes highly vascular. The walls of the congested arterioles and capillaries undergo degenerative changes which increase their porosity and lessen their resistance, thus favoring diapedesis and rupture. Blood is poured out into the interglandular connective-tissue and forces its way through the epithelial barriers into the *cavum uteri,* or accumulates in the deeper areolar spaces. Because of this mechanical violence and interfrence with its food-supply, the over-lying mucous membrane undergoes fatty degeneration and is thrown off in the form of granular and shreddy tissue-debris, to be regenerated during the later reparative stage.

The degree of congestion incident to menstruation varies just as in inflammation. In the case of a young girl who is nearing puberty, a periodical white mucous discharge attests the mild congestion of the uterine mucosa which leads only to the migration of leucocytes and to glandular stimulation; while, after maturity is reached, the more intense process is evidenced by the presence not merely of mucus and glandular secretions but of blood, together with the histological components of the destroyed tissues.

The symptoms which usually accompany menstruation are local and reflex in character. There is a feeling of fullness, weight and pressure in the lower abdomen and pelvis, due to the congestion and greater bulk of uterus and adnexa. The individual is subject to headache, frequently severe in character. She is inclined to be nervous, irritable and hypersensitive to her physical and mental impressions. The skin is prone to disorders of a congestive, pigmentary and eruptive character; while the breasts, thyroid, parotid and tonsils are apt to increase in size, influenced probably by ovarian hormones.

In the normal girl or woman, however, menstrual pain should be absent. In fact, many individuals, particularly during the period of adolescence, do not feel called upon to interrupt their usual rounds of duty and pleasure because of this functional disturbance, which, to them, is objectionable for reasons purely esthetic in character. Again, extensive disease of the genital organs, serious and even fatal in character, may be present and yet occasion no dysmenorrhoea.

As a rule, however, painful menstruation is due to pathological conditions, either local or general in character; though the casual factor or factors may not be demonstrable. During the menstrual molimen a woman may undergo physical torture which necessitates recourse to powerful anodynes, even anesthetics; and yet a most painstaking examination discloses little or no pathological change to account for her suffering. In the great majority of cases, however, such conscientious diagnostic efforts are rewarded by the discovery of local or systemic conditions which afford the needed information. Ofter definite abnormalities are easily made out; and, if these are curable, we can promise our patients the desired relief.

The pains which characterize dysmenorrhoea do not include the general *malaise,* with pelvic weight and fullness which, as we have already noted, are symptomatic of normal menstruation. They are rather described as dull, heavy, sharp, dragging, radiating or bearing-down; and their usual location is in the loins, or low down in the back or pelvis. Some women complain of their suffering during the whole period; in others the pains begin or end with the establishment of the flow. In my own experience, the time of maximum pain with reference to the appearance of the discharge is of great importance from a diagnostic point of view. Thus, in patients whose dysmenorrhoea is conterminous with the menstrual period, I am disposed to look for systemic, or widely diffused local, trouble. When the pains antedate the establishment of the flux, the ovaries or tubes, or both, are usually at fault; while the cause of the later suffering is generally to be looked for in the uterus and lower genital tract.

The pains of dysmenorrhoea, as in all other conditions, are due to stimulation of the sensory nerves, the afferent feeders, as it were, of the cerebro-spinal nerve-centers. Painful impulses are transmitted from the genital organs partly through the myelinic, or white, dendrons which reach the cord through the white rami communicantes, partly through the afferent fibres of the third and fourth sacral nerves, which join the pelvic, or inferior, offsets of the hypogastric plexus directly from the anterior primary divisions of these nerves as visceral branches, without the mediation of the gangliated cord of the sympathetic. Entering the cord through the ganglia and fibres of the posterior roots these impulses, as do those of heat and cold, ascend through the gray matter of the cord to the optic thalamus, whence they reach the sensory cerebral cortex through fibres of the corona radiata. Muscular and tactile sensations are transmitted to the cortex via the posterior columns of white matter.

Pathological confirmation of these facts is afforded by the diseases known as syringomyelia and locomotor ataxia. In the former, a disease of the gray matter about the central spinal canal, conscious perception of painful and thermal sensations is lost, while tactile impulses reach the cortex. In the latter disease, which involves the afferent neurons and the posterior columns of Goll and Burdach, pain, heat and cold are felt while muscular and tactile sensations are no longer experienced.

The muscular coats of the ovaries, tubes, uterus and vagina, as far as the hymen, are made up of smooth muscle-fibres, the hymen representing the more or less intact partition between the vagina and the uro-genital sinus, which latter constitutes the floor of the pudendal cleft from the clitoris to the fossa navicularis. These involuntary fibres, as well as the peritoneum, blood-vessels and glands of the genital organs are under the control of the splanchnic or sympathetic nerves primarily, though linked to the central nervous system in the manner already described.

Under normal conditions afferent impulses from these organs do not raise to consciousness, even pain is often absent on injury. When the irritation is very great, however, pain is referred to areas of skin supplied by those somatic nerves whose visceral branches are thus affected. In this way the pains incident to dysmenorrhoea, affecting the back and loins, are accounted for. Viscera which are innervated purely by sympathetic nerve-fibres are insensitive, the presence of somatic nerve-branches being necessary for the reception of pain and other sensations. For this reason the anterior margin of the liver, the gall-bladder, stomach, intestine and mesentery may be cauterized or cut without subjective pain; while the parietal peritoneum is alive to painful impressions but not to touch. If we bear in mind the connecting links between the central and visceral nervous systems, the symptom-complex associated with dysmenorrhoea is easily explained. Thus, an infective process involving the pelvic visceral peritoneum may be attended with vomiting, abdominal rigidity, paresis and other reflex symptoms, as well as pain. The pressure of inflammatory exudates upon the sensory nerve-endings, and perhaps their chemical irritation, are largely responsible for the condition known as dysmenorrhoea.

The lesions associated etiologically with painful menstruation may be grouped into two classes: Local and General.

The local conditions commonly present are: (1) Inflammation, (2) Tumors, (3) Obstruction and (4) Malformation and defective development.

Among the general or systemic causes, acting alone or in conjunction with local lesions, are anaemia, chlorosis, gout, rheumatism, hysteria and neurasthenia (Dudley).

The key to the rational treatment of dysmenorrhoea is accuracy of diagnosis. Is the pain in a given case referable to a local or general condition, or to both combined? This query having been correctly solved, the treatment, whether medical or surgical, conservative or radical, palliative or curative, will suggest itself; and upon the good judgment, as well as upon the medical and surgical ability of the attendant will depend a satisfactory outcome.

In view of the wide range of the subject, it is obviously impossible to give a detailed enumeration and description of the therapeutic measures to be adopted for the relief or cure of dysmenorrhoea in all its phases. We must rather content ourselves, in conclusion, with a few observations based upon the experiences of the past. In the first place, radical measures should be instituted only after careful deliberation and with guarded prognoses. Major operations are often indicated in cases of infective inflammations and tumors, involving any or all of the generative organs, and an associated dysmenorrhoea is thereby usually relieved. In some instances, however, the pain is referred to one or both ovaries. Combined palpation may disclose exquisite tenderness of these organs, and the urgency of the neurotic symptom may be so great as to lead to operative intervention. Exposed to view, however, these organs may be the seat of no demonstrable lesions; and, under such circumstances, their removal seldom affords the desired relief.

The great majority of patients who consult physicians for painful menstruation are the victims of uterine displacements and abnormal flexures, coupled, ofttimes, with metritis and endometritis of varying severity. In such cases, generally young unmarried, or childless women, the pain is chiefly referable to mechanical causes. Unnatural pressure upon the rectum and sacral nerves, or pressure and dragging upon the bladder, play their respective parts as algetic agencies.

Greater than these, however, is the mechanical interference with the blood-flow incident to such conditions. Uterine flexions may be, and are, responsible for the more or less complete occlusion of the uterine canal at the point of bending and a resultant obstructive dysmenorrhoea. The same conditions, however, interfere with the local patency of the vessels, particularly the veins, and initiate all the evils accompanying congestion of the passive type. The uterine tissues are more or less devitalized from lack of a normal food-supply, and atrophy accordingly. Or, the organ becomes congested, swollen and infiltrated with exudates. Connective-tissue hyperplasia may ensue; and, from the pressure and chemical irritation due to these conditions, the sensory nerves bear their tales of woe to the cerebral cortex and urge the sufferer to seek the aid of her physician.

Fortunately, dysmenorrhoea which is occasioned by these most common conditions is easily cured or abated. For the displacements, tampons, properly selected and properly fitted pessaries and the Alexander operation or its internal modifications will prove effective in the great majority of cases. For the menstrual pains due to uterine flexures and their attendant changes in the endometrium simple instrumental dilatation and curettage, properly performed, usually afford great and lasting improvement, often a cure. Stem pessaries have been strongly advocated by many physicians of large experience in the treatment of these angulate canals, but the fear of evil consequences has tended to limit their use.

In extreme cases of flexion the cervix may be incised in a manner best suited to the individual condition, the object being to straighten and enlarge the cervical canal. Bilateral incision of the cervix, hollowing out the intermediate tissues and uniting the vaginal and inner cervical edges with interrupted or continuous catgut sutures is a very effective proceeding, usually accredited to Pozzi. Not only is the dysmenorrhoea thereby relieved but conception is facilitated, and the familiar curative effect of gestation and parturition may be utilized.

Medical relief, often of a very satisfactory character, may result from the use of remedies best adapted to any abnormal systemic conditions which may be present, coupled with the palliative use of phenalgin, antikamnia and other coal-tar derivatives, aspirin and codeia. Used singly or in suitable combinations, these drugs are well adapted to such cases as refuse to undergo the more or less trying ordeals incident to surgical procedures.

In prescribing for this class of sufferers, however, the dangers ever incident to the palliative treatment of pain should be borne in mind. Thoughtless approval of alcoholic aids and the exhibition of various insidious drugs, unaccompanied with suitable warning and advice, are highly reprehensible in the treatment of dysmenorrhoea, because of its recurrent nature. Bitter indeed must be the reflections of a physician who is forced to realize that an innocent woman has been added to the pitiful host of drunkards and drug-fiends through his instrumentality.

164 FRANKLIN STREET.

